# Meta-analysis comparing direct oral anticoagulants versus vitamin K antagonists in patients with left ventricular thrombus

**DOI:** 10.1371/journal.pone.0252549

**Published:** 2021-06-04

**Authors:** Kazuhiko Kido, Yasir Abdul Ghaffar, James C. Lee, Christopher Bianco, Mikiko Shimizu, Tsuyoshi Shiga, Masayuki Hashiguchi

**Affiliations:** 1 Department of Clinical Pharmacy, West Virginia University School of Pharmacy, Morgantown, WV, United States of America; 2 Division of Cardiology, Department of Medicine, West Virginia University, Morgantown, WV, United States of America; 3 Department of Pharmacy Practice, University of Illinois Chicago College of Pharmacy, Chicago, IL, United States of America; 4 Department of Pharmaceutics and Pharmacometrics, School of Pharmacy, Shujitsu University, Okayama, Japan; 5 Department of Clinical Pharmacology and Therapeutics, The Jikei University School of Medicine, Tokyo, Japan; 6 Division for Evaluation and Analysis of Drug Information, Faculty of Pharmacy, Keio University, Tokyo, Japan; Leiden University Medical Center, NETHERLANDS

## Abstract

Current American College of Cardiology/American Heart Association guidelines for stroke or ST-elevation myocardial infarction recommend the use of oral vitamin K antagonists (VKAs) as a first-line anticoagulant. Although several studies have compared the use of direct oral anticoagulants (DOACs) to VKAs for left ventricular thrombus (LVT) anticoagulation therapy, they are small scale and have produced conflicting results. Thus, this meta-analysis was performed to aggregate these studies to better compare the efficacy and safety of DOACs with VKAs in patients with LVT. Cochrane Library, Google Scholar, MEDLINE, and Web of Science database searches through January 10, 2021 were performed. Eight studies evaluating stroke or systemic embolism (SSE), six studies for LVT resolution, and five studies for bleeding were included. There were no statistically significant differences in SSE (OR 0.89; 95% CI 0.46, 1.71; p = 0.73; *I*^2^ = 45%) and LVT resolution (OR 1.13; 95% CI 0.75, 1.71; p = 0.56; *I*^2^ = 1%) between DOAC and VKA (reference group) therapy. DOAC use was significantly associated with lower bleeding event rates compared to VKA use (OR 0.61; 95% CI 0.40, 0.93; p = 0.02; *I*^2^ = 0%). DOACs may be feasible alternative anticoagulants to vitamin K antagonists for LV thrombus treatment. Randomized controlled trials directly comparing DOACs with VKAs are needed.

## Introduction

Left ventricular thrombus (LVT) development is common in patients with severe left ventricular (LV) dysfunction, often in the setting of acute anterior wall myocardial infarction (MI) and nonischemic cardiomyopathies, and is associated with increased risk of stroke or systemic embolism (SSE) [1–3]. Patients with cardioembolic stroke are at highest risk of in-hospital mortality during the acute phase, with subsequent long-term disability following the initial course [4,5]. Pre-requisites for LVT formation include endothelial injury, hypercoagulability, and venous stasis, (i.e., Virchow’s triad), and can occur as early as within 24 hours to 3 months following MI [1]. Additionally, the potential for LVT cerebral embolization persists in patients who develop chronic LV dysfunction. In heart failure with reduced ejection fraction (HFrEF), a hypercoagulable state is noted with increased incidence of LVT and higher risk of thromboembolism [6].

Current American College of Cardiology Foundation/American Heart Association (ACCF/AHA) guidelines recommend the use of oral vitamin K antagonists (VKAs) as the primary anticoagulant in the management of ST-elevation myocardial infarction and asymptomatic LV mural thrombi (Class IIa, Level of Evidence C) [7]. The European Society of Cardiology (ESC) more specifically recommends 6 months of oral anticoagulant therapy with VKA (Class IIa, Level of Evidence C) [8]. Achieving adequate anticoagulation with warfarin requires medication adherence, dietary consistency, frequent laboratory monitoring, and a narrow time in therapeutic range (TTR). These disadvantages have led to increased adoption of direct oral anticoagulants (DOACs) for anticoagulation treatment of thromboembolic diseases such as atrial fibrillation (AF) and venous thromboembolism (VTE) [9].

DOAC use for anticoagulation for LVT in patients with LV dysfunction and STEMI remains controversial. LVT formation is pathologically similar to left atrial appendage thrombus (LAAT) formation due to a low-flow and low-shear setting, with DOAC use for left atrial appendage thrombi (LAAT) appearing to be highly efficacious [10]. Correspondingly, the safety and efficacy of DOACs observed in the prevention of thromboembolism and stroke in atrial fibrillation have served as a basis for DOAC use for LVT anticoagulation [11–14]. Since the publication of ACC/AHA guidelines in 2013, multiple studies and case series have compared DOACs with VKAs in patients with LVT but have produced discordant results. Despite the suggestion of generally improved safety profile of DOACs over VKA in other anticoagulation contexts, the safety and efficacy of DOACs for LVT anticoagulation remain inconclusive due to inconsistent trial outcomes thus far [15]. Thus, this meta-analysis of published full-text clinical study manuscripts comparing DOACs to VKAs in LVT was performed to better compare the efficacy and safety of DOACs with VKAs in patients with LVT, specifically SSE, LVT resolution, and overall bleeding.

## Methods

Literature search keywords utilized for the MEDLINE search included: ((left ventricular thrombus) or (left ventricular thrombi)) and ((direct oral anticoagulant) or apixaban or dabigatran or edoxaban or rivaroxaban)). No limit was used for database searches. Database searches through January 10, 2021 were performed using the Cochrane Library, Google Scholar, MEDLINE, and Web of Science. Two independent investigators (KK, MH) performed the literature search and selected articles based on pre-specified inclusion and exclusion criteria. Inclusion criteria included: 1. patient age >18 years old diagnosed with LVT and 2. clinical studies comparing DOACs with VKAs. Conference abstracts were excluded. Studies were excluded if they were case series, non-English articles, or studies not evaluating VKAs.

Two independent investigators (KK, JL) extracted baseline characteristics, SSE, LVT resolution, bleeding outcome results, follow-up period, and number of subjects. The primary efficacy outcome was SSE event rate, and the primary safety outcome was bleeding. The secondary efficacy outcome was LVT resolution rate. Major bleeding was not evaluated since this was not evaluated in the majority of included studies. Study quality was evaluated using the Newcastle-Ottawa Scale (NOS) [16].

A random-effects model was selected, and a fixed effects model was used for sensitivity analysis. Heterogeneity was assessed with *I*^2^ statistics. Publication bias was assessed with the Egger regression test. An odds ratio (OR) and 95% confidence interval (CI) were estimated, and p-values < 0.05 were defined as statistically significant. The Preferred Reporting Items for Systematic Reviews and Meta-analysis guidelines were followed to conduct this meta-analysis [17]. All results were analyzed with RevMan 5.3 (Nordic Cochrane Centre, Cochrane Collaboration, Copenhagen). The protocol was not registered.

## Results

**Fig 1:**
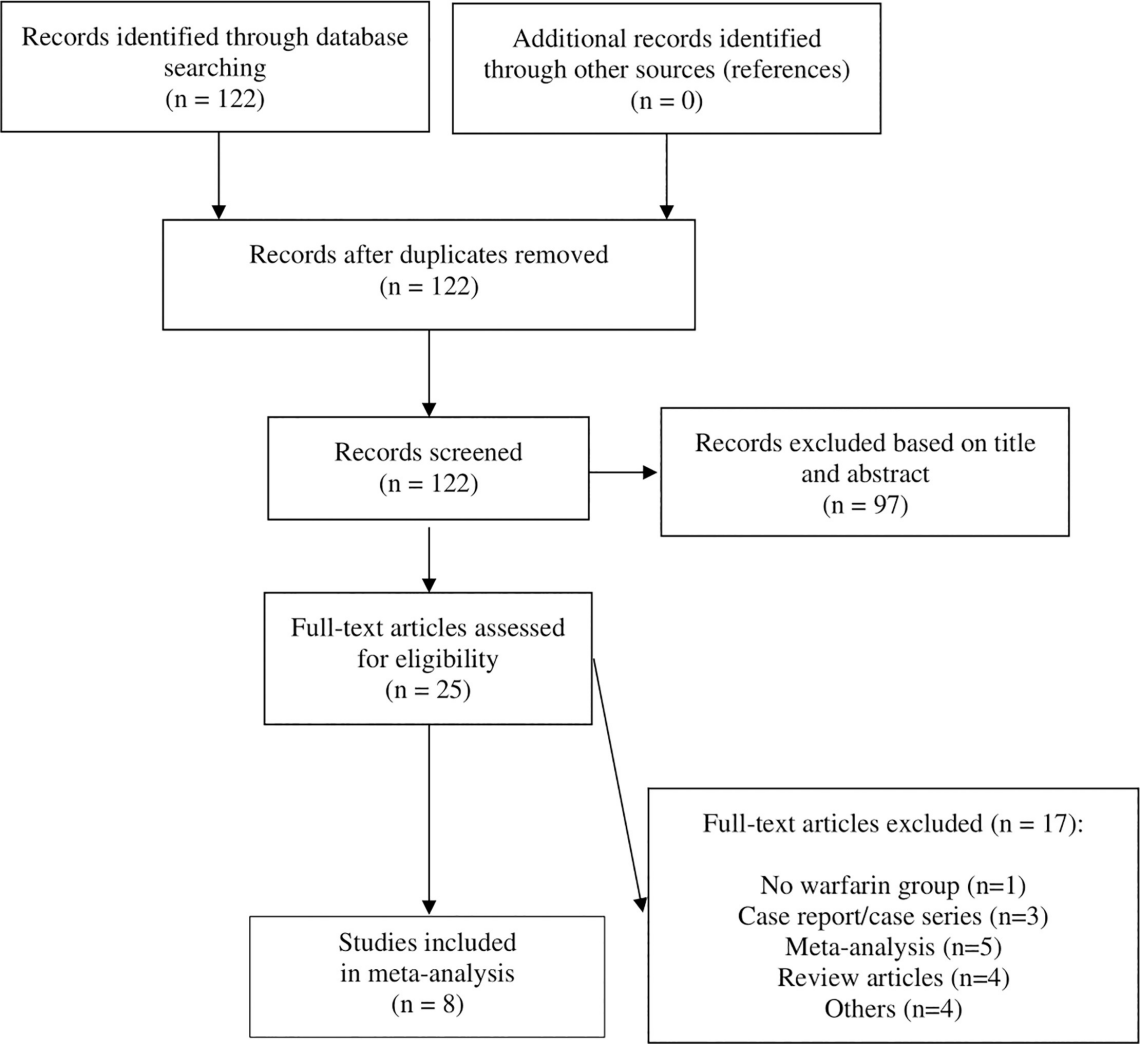
Flow diagram of DOACs versus VKA in patients with left ventricular thrombus literature search.

A total of 122 articles were evaluated for eligibility, and eight studies were included for the final analysis (Fig 1) [11–13,15,18–21]. One study was excluded because the comparison of VKA with DOACs was not the primary study purpose and less than 5 patients on DOACs were included [10].

Tables 1 and 2 describe the key baseline characteristics, follow-up period, and outcomes of the included studies. All included studies evaluated SSE, 6 studies evaluated LVT resolution, and 5 studies evaluated bleeding. NOS quality assessment results are included in Supporting Information (S1 Table). A total of 454 patients in the DOAC group and 1438 patients in the VKA group were evaluated.

**Table 1:** Key baseline patient characteristics of included studies.

Study	Age (years, mean ± SD or [IQR])	Sex (%, male)	SCr (mg/dL) or eGFR (mL/min/1.73m^2^)	LVEF (% ± SD or [IQR])	ICM (%) or MI %	HTN (%)	DM (%)	HLD (%)	AF (%)	Antiplatelet therapy (%)
Daher et al.	DOAC vs. VKA 57±14 vs. 61±13	82.4 vs. 83.0	NR	41±8 vs. 36± 12	ICM 88 vs. 74	59 vs. 40.5	12 vs. 21.4	29.4 vs. 43	NR	NR
Robinson et al.	DOAC vs. warfarin 58.1±14.9 vs. 58.2±15.1	77.7 vs. 72	eGFR 80.5±29.3 vs. 75.8±29.8	27.7±13.8 vs. 28.2±12.4	ICM 54.5 vs. 62.7	71.1 vs. 75	29.8 vs. 39	58.7 vs. 53.4	24.8 vs. 19.1	63.6 vs. 69.5
Ali et al.	DOAC vs Warfarin 58.2±11.9 vs. 58.1±16.3	81.3 vs. 81.7	NR	23±9.4 vs. 23.2±11.2	Overall: ICM 58% Acute MI 15%	NR	37.5 vs. 30	NR	28.1 vs. 30	Overall: ASA 65.45; CLOP 14.5; TIC 0.91; PRAS 1.82
Guddeti et al.	DOAC vs Warfarin 60.7±13.1 vs. 61.3±12.2	79.0 vs. 68.8	NR	25 [20–40] vs. 25 [25–35]	ICM 52.6 vs. 60 MI 52.6 vs. 56.4	79.0 vs. 76.3	15.8 vs. 43.0	NR	21.1 vs. 22.5	ASA 57.9 vs. 67.5; P2Y12i: 15.8 vs. 15
Iqbal et al.	DOAC vs VKA 62±13 vs. 62±14	91 vs. 89	1.13±0.23 vs. 1.03±0.35	31+13 vs. 35+13	ICM 82 vs. 89	41 vs. 29	86 vs. 31	18 vs. 15	14 vs. 5	ASA 41 vs 65; CLOP 50 vs. 35; TIC 0 vs. 10
Willeford et al.	DOAC vs. Warfarin 54 [48–64] vs. 56 [49–65.5]	77.4 vs. 80.6	NR	NR	MI 22.7 vs. 26.4	41.9 vs. 36.4	18.2 vs. 28.7	NR	13.6 vs. 18.6	ASA 22.7 vs. 54.3; P2Y12i: 13.6 vs. 27.9
Jones et al.	DOAC vs. Warfarin 58.73±14.2 vs. 60.81±14.3	80.4 vs. 85	eGFR 68.24±15.8 vs. 66.11±18.8	33.5+10.0 vs. 35.4+9.0	Prior MI* 55.3 vs. 36.7	60.5 vs. 36.4	18.4 vs. 16.7	50 vs 31.7	NR	Single antiplatelet: 24.4 vs 21.7; Triple therapy 68.3 vs 70.0
Bass et al.	DOAC vs. warfarin 63.4±16.7 vs 61.6±15.3	69.4 vs. 70.9	SCr 1.00 ± 0.38 vs. 1.33 ± 1.12	NR	MI 42.8 vs. 57.6	NR	NR	NR	61.7 vs. 45.8	46.7 vs. 55.7

AF: Atrial fibrillation; ASA: Aspirin; CLOP: Clopidogrel; DOAC: Direct oral anticoagulant; DM: Diabetes, eGFR: Estimated glomerular filtration rate; HTN: Hypertension; HLD: Hyperlipidemia; ICM: Ischemic cardiomyopathy; IQR: Interquartile range; LV: Left ventricular; LVEF: Left ventricular ejection fraction; MI: Myocardial infarction; NR: Not reported; P2Y12i: P2Y12 inhibitor; TIC: Ticagrelor; VKA: Vitamin K antagonist.

* The patient population of this study were patients presenting with acute MI undergoing primary percutaneous coronary intervention.

**Table 2:** Key study design characteristics and results of included studies.

Study design	Subjects (# patients, unless otherwise stated)	Follow-up period [IQR]	Efficacy outcome (DOAC vs. VKA)	Safety outcome (DOAC vs. VKA)
Daher et al. Single-center retrospective cohort study	DOAC 17 (API 12, RIV 4, DAB 1) vs. VKA 42	NR	SSE: 11.8% vs. 9.5% LV thrombus resolution at 3 months: 70.6 vs. 71.5%	NR
Robinson et al. Multi-center retrospective cohort study	DOAC 121 (API > RIV > DAB) vs. warfarin 236	Median 351 days [51–866)	SSE at 1 month: 14.0% vs. 5.9% (p = 0.01)	Bleeding: 6.6% vs. 8.1%
Ali et al. Single-center retrospective cohort study	DOAC 32 (API 13; RIV 18; DAB 1) vs. warfarin 60	<1 year: 24.6% 1–3 years: 22.7% 3–5 years: 17.3% >5 years: 18.2%	SSE: 6% vs 26.6% LV thrombus resolution: 53% vs. 62%	Bleeding: 0 vs. 3.3% (hemorrhagic CVA)
Guddeti et al. Multi-center retrospective cohort study	DOAC 19 (API 15; RIV 2; DAB 2) vs. warfarin 80	Mean 10.4±3.4 months Median 1 year	Ischemic stroke at 1 year: 0 vs. 2.5% LV thrombus resolution: 80% vs. 81%	Bleeding: 5.3% vs 6.25%
Iqbal et al. Single-center retrospective cohort study	DOAC 22 (API 8; RIV 13; DAB 1) vs. warfarin 62	Mean 3.0±1.4 years	Thromboembolic events: 0 vs. 2% LV thrombus resolution: 65% vs 76% All-cause mortality: 14% vs. 10% Repeat hospitalization: 45% vs. 50%	Clinically relevant bleeding: 0 vs. 10%
Willeford et al. Single-center retrospective cohort study	DOAC 22 (API 4, RIV 18) vs. warfarin 129	Median 254 days [98–343]	Composite of LV thrombus persistence and SSE: 40.9% vs. 54.3%	Composite of hemorrhagic stroke or bleeding requiring transfusion: 4.5% vs. 3.9%
Jones et al. Single-center retrospective cohort study	DOAC 41 (API 36.5%, RIV 58.5%, EDO 5%) vs. warfarin 60	Median 2.2 years	LV thrombus resolution at 1 year: 82% vs. 64.4% (p = 0.0018) SSE: 2.4% vs. 5%	Bleeding BARC >2: 0% vs. 6.7%
Bass et al. Multi-center retrospective cohort study	DOAC 180 (API 79, RIV 77, DAB 29) vs. warfarin 769	NR	Thromboembolic stroke at 90 days: 7.8% vs. 11.7% SSE: 33% vs. 30.6%	GUSTO bleeding 10.9% vs. 7.8% Blood product administration: 25.8% vs. 13.9% (p<0.001)

API: Apixaban; DAB: Dabigatran; DOAC: Direct oral anticoagulant; EDO: Edoxaban; GUSTO: Global Use of Strategies to Open Coronary Arteries; IQR: Interquartile range; LV: Left ventricular; NOS: Newcastle-Ottawa scale; NR: Not reported; RIV: Rivaroxaban; SSE: Stroke or systemic embolism; VKA: Vitamin K antagonist.

**Fig 2:**
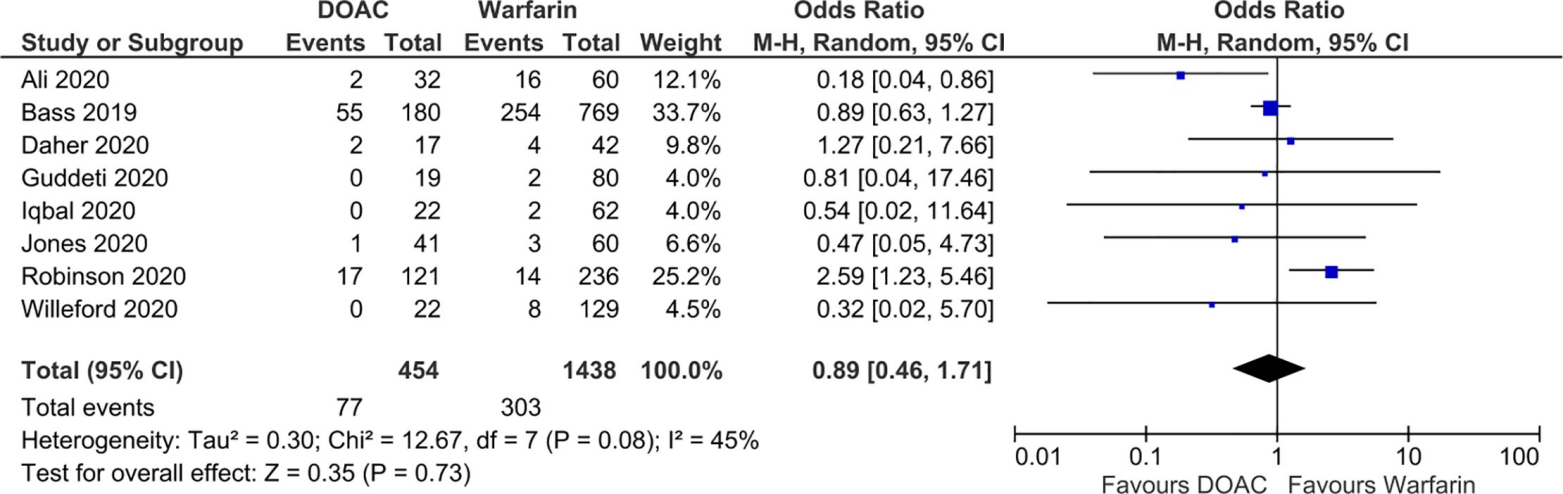
Forest plot of stroke of systemic embolism event rate in patients with left ventricular thrombus receiving DOACs versus VKA.

**Fig 3:**
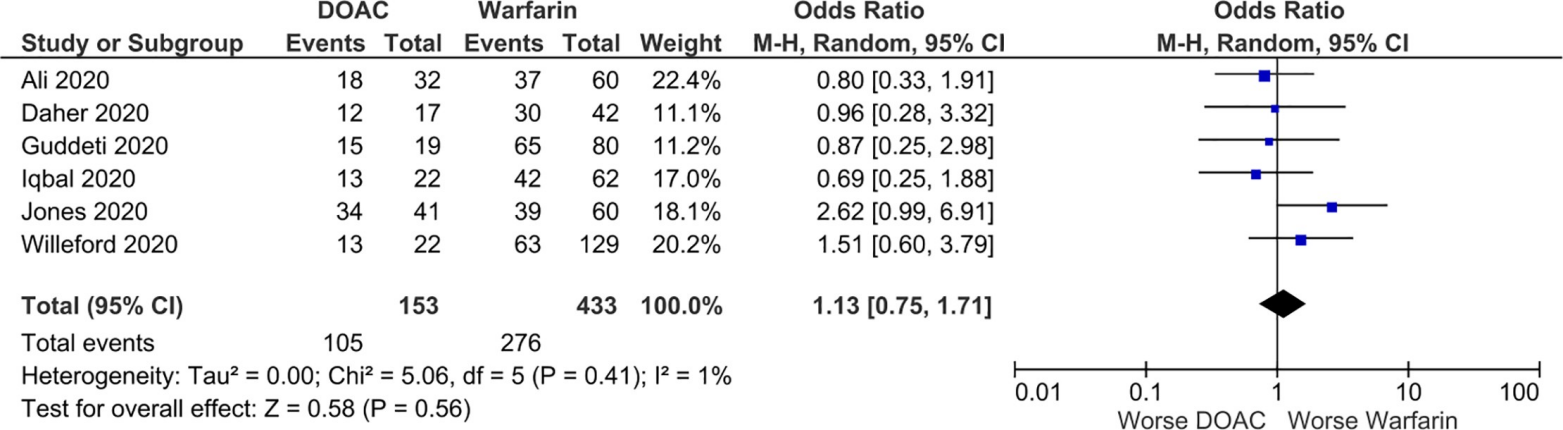
Forest plot of left ventricular thrombus resolution rate in patients with left ventricular thrombus receiving DOACs versus VKA.

**Fig 4:**
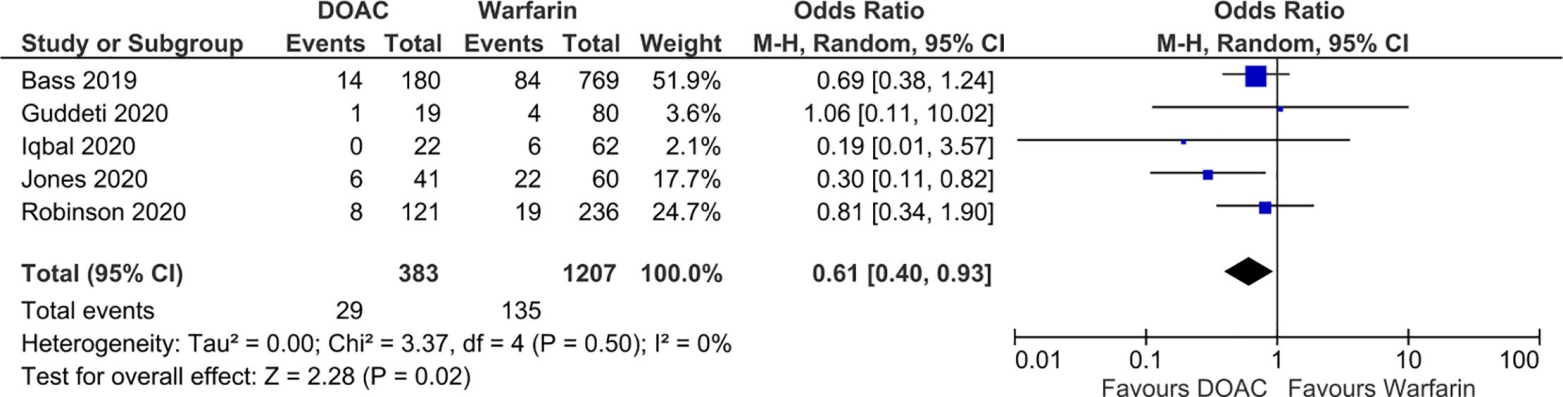
Forest plot of bleeding event rate in patients with left ventricular thrombus receiving DOACs versus VKA.

There were no significant differences in SSE (OR 0.89; 95% CI 0.46, 1.71; p = 0.73; *I*^2^ = 45%) and LVT resolution (OR 1.13; 95% CI 0.75, 1.71; p = 0.56; *I*^2^ = 1%) between the DOAC and VKA (reference group) groups (Figs 2 and 3). DOACs were associated with significantly lower bleeding event rates compared to VKA (OR 0.61; 95% CI 0.40, 0.93; p = 0.02; *I*^2^ = 0%) (Fig 4). The fixed effects model found no significant difference in SSE (OR 0.94; 95% CI 0.70, 1.25; p = 0.66; *I*^2^ = 45%) or LVT resolution (OR 1.15; 95% CI 0.77, 1.73; p = 0.50), and DOAC was still associated with significantly lower bleeding event rates compared to VKAs (OR 0.60; 95% CI 0.39, 0.91; p = 0.02; *I*^2^ = 0%). No significant funnel plot asymmetry was found by the Egger regression test, indicating no significant publication bias (SSE: p = 0.67; LVT resolution: p = 0.75; bleeding: p = 0.91).

## Discussion

This meta-analysis found DOAC use for the anticoagulation treatment of LVT had comparable SSE and LVT resolution rates to warfarin. Additionally, patients treated with DOACs experienced significantly lower bleeding events compared to warfarin. The SSE and LVT resolution outcomes of this meta-analysis are congruent with the outcomes of other recently published meta-analyses [22–27]. Unlike previous meta-analyses, however, this meta-analysis only included fully published manuscripts and did not include abstracts due to their preliminary nature, which likely reduced the chance of error for data extraction [22,24]. Especially, fully published data from the biggest included study by Bass et al. was included in our meta-analysis but other meta-analyses only included abstract data.

Notably and contrary to our findings, however, Robinson et al observed increased SSE rates with DOAC use compared to warfarin, although these events occurred late in the course of treatment when survival curves began to diverge [15]. Several plausible explanations exist. First, although treatment switching was allowed between the study groups, increased SSE risk persisted even after adjusting for several confounders in the multivariate analysis [15]. Second, this outcome may have been influenced by the DOAC studied. Apixaban was the predominant DOAC used (76.2% of participants), possibly due to insurance coverage or local and regional practice variations [28]. Third, the use of both reduced and standard apixaban doses (2.5 mg vs 5mg twice a day) and anticoagulation therapy interruption during acute hospitalizations and post discharge following LVT diagnosis may have led to the later emergence of strokes [29]. In response, Robinson et al reported only 4 of 73 (5%) patients treated with apixaban were treated with the low-dose regimen and none developed stroke and that all anticoagulation interruptions were accounted for in the time dependent analysis. Despite these adjustments, DOAC use remained a predictor of SSE [30].

With respect to LVT resolution, a recent large meta-analysis of patients taking DOACs observed thrombus resolution in only 80% of patients [31]. Patients anticoagulated for LVT may remain at increased risk of thromboembolism and continued presence of unresolved left ventricular thrombi despite complete initial thrombus resolution [32]. Previous data have also suggested a persistent risk of thromboembolism despite LVT resolution, with one platelet imaging study demonstrating externally detectable ongoing platelet accumulation indicating continued surface activity [33]. At this time, it remains unclear if duration of anticoagulation therapy should extend beyond 3 months and which DOAC dose is the most appropriate for LVT anticoagulation (i.e. DOAC doses approved for AF or doses for venous thromboembolism and whether initial overlap with parenteral anticoagulation is necessary) [28].

The combination of dual antiplatelet therapy with oral anticoagulation (i.e. triple antithrombotic therapy) also remains an issue for patients with LVT and is associated with high rates of fatal and nonfatal bleeding complications [34]. One network meta-analysis found the use of a DOAC plus P2Y12 inhibitor two-drug regimen was associated with lower bleeding compared with VKA and P2Y12 inhibitor [35]. Despite enrolling a large number of patients on triple antithrombotic therapy (68.3% DOAC vs 70.0% VKA), Jones et al found less major bleeding with DOAC use compared to VKA (0% vs 6.7%, p = 0.030) [18]. Bass et al, on the other hand, observed comparable bleeding events (10.9% vs 7.8%, p = 0.40) between warfarin and DOAC therapy, although more warfarin patients received blood products compared to those taking a DOAC (25.8% vs 13.9%, p < 0.001) [11].

Formation of an LV thrombi mirrors that of LAAT and occurs in a low-flow and low-shear environment. This contrasts with thrombus formation with mechanical heart valves, which is predominantly contact-pathway mediated and where DOAC use has appeared to be inferior to warfarin [36]. When considering the similarities in thrombus formation and the efficacy of DOAC use in atrial fibrillation treatment, however, it is reasonable to conject similar efficacy when used in the treatment of LV thrombi. Additionally, DOACs may achieve more consistent anticoagulant effects and up to 50% reduced risk of intracranial hemorrhage compared to VKAs [37]. The favorable pharmacologic and clinical profile of DOACs will undoubtedly make their selection over warfarin for anticoagulation therapy attractive in patients with known or suspected LV thrombus.

### Limitations

First, despite the large number of patients analyzed in this study, the overall number of outcome events was relatively modest, yielding wide CIs and increasing the risk of type II error. Second, the studies included were all observational studies, and consequently, endpoint ascertainment and classification were likely to vary according to each study’s definition. Third, this analysis did not have enough sample size to investigate the efficacy and safety of an individual DOAC compared to warfarin. Finally, DOAC dose and the duration of treatment for LV thrombus were not investigated in this study.

## Conclusions

In this meta-analysis of published observational LVT anticoagulation full-text study data, there were no differences in stroke or systemic embolism and left ventricular thrombus resolution between direct oral anticoagulant and warfarin therapy. DOAC use was associated with significantly less bleeding compared to warfarin. Prospective, randomized clinical trials are needed to confirm the safety and efficacy of DOACs for the use of left ventricular thrombus anticoagulation.

## Supporting information

S1 Checklist PRISMA 2009 checklist Kido. (DOC)

S1 Table Newcastle-Ottawa quality assessment of the included studies. (DOCX)

S1 Dataset Dataset LV thrombus DOAC vs. Warfarin. (XLSX)
