# Supplementary material for: Meta-analysis comparing direct oral anticoagulants versus vitamin K antagonists in patients with left ventricular thrombus
Source: PLoS One. 2021 Jun 4;16(6):e0252549. doi: 10.1371/journal.pone.0252549 (PMC8177531; doi:10.1371/journal.pone.0252549)
Supplement: S1 Table — (DOCX) [file pone.0252549.s002.docx]

Supplemental table 1. Newcastle-Ottawa quality assessment of the included studies

|  | Daher 2020 | Robinson 2020 | Ali 2020 | Guddeti 2020 | Iqbal 2020 | Willeford 2020 | Jones 2020 | Bass 2021 |
| --- | --- | --- | --- | --- | --- | --- | --- | --- |
| **Selection** | **4 stars** | **4 stars** | **4 stars** | **4 stars** | **4 stars** | **4 stars** | **4 stars** | **4 stars** |
| Representativeness of the exposed cohort | 1 star (somewhat representative of the average cohort in the U.S.) | 1 star (somewhat representative of the average cohort in the U.S.) | 1 star (somewhat representative of the average cohort in the U.S.) | 1 star (somewhat representative of the average cohort in the U.S.) | 1 star (U.K. cohort, somewhat representative of the average cohort in the U.S.) | 1 star (somewhat representative of the average cohort in the U.S.) | 1 star (U.K. cohort in primary PCI, somewhat representative of the average cohort in the U.S.) | 1 star (somewhat representative of the average cohort in the U.S.) |
| Selection of non-exposed cohort | 1 star (drawn from the same cohort as the exposed cohort) | 1 star (drawn from the same cohort as the exposed cohort) | 1 star (drawn from the same cohort as the exposed cohort) | 1 star (drawn from the same cohort as the exposed cohort) | 1 star (drawn from the same cohort as the exposed cohort) | 1 star (drawn from the same cohort as the exposed cohort) | 1 star (drawn from the same cohort as the exposed cohort) | 1 star (drawn from the same cohort as the exposed cohort) |
| Ascertainment of exposure | 1 star (medical record) | 1 star (medical record) | 1 star (medical record) | 1 star (structured data collection form) | 1 star (medical record) | 1 star (medical record) | 1 star (prospective database) | 1 star (medical record) |
| Demonstration that outcome of interest was not present at start of study | 1 star (yes) | 1 star (yes) | 1 star (yes) | 1 star (yes) | 1 star (yes) | 1 star (yes) | 1 star (yes) | 1 star (yes) |
| **Comparability** | **0 stars** | **2 stars** | **0 stars** | **0 stars** | **0 stars** | **2 stars** | **2 stars** | **2 stars** |
| Comparability of cohorts based on the design or analysis | 0 stars (the study does not control for any variable) | 2 stars (the study controls for multiple variables) | 0 stars (the study does not control for any variable) | 0 stars (the study does not control for any variable) | 0 stars (the study does not control for any variable) | 2 stars (the study controls for multiple variables) | 2 stars (the study controls for multiple variables) | 2 stars (the study controls for multiple variables) |
| **Outcome** | **3 stars** | **3 stars** | **3 stars** | **3 stars** | **3 stars** | **3 stars** | **3 stars** | **3 stars** |
| Assessment of Outcomes for Included Cohort Studies | 1 star (medical records) | 1 star (medical records) | 1 star (medical records) | 1 star (structured data collection form) | 1 star (medical records) | 1 star (medical records) | 1 star (medical records) | 1 star (medical records) |
| Was follow-up long enough for outcomes to occur? | 1 star (Yes. At least 3 months of anticoagulation therapy) | 1 star (Yes) | 1 star (Yes, 75.4% patients with follow-up >1 year) | 1 star (Yes. Average 10.4 months) | 1 star (Yes. Average 3 years; average 677 days of anticoagulation therapy | 1 star (Yes. Median 254 days) | 1 star (Yes. Median 2.2 years; at least 6 months of anticoagulation therapy) | 1 star (Yes; 90 days) |
| Adequacy of cohort follow-up | 1 star (all patients received anticoagulation for at least 3 months) | 1 star (subjects lost to follow-up unlikely to introduce bias. Sensitivity analysis supports retained results based on duration of anticoagulation) | 1 star (subjects lost to follow-up unlikely to introduce bias.) | 1 star (subjects lost to follow-up unlikely to introduce bias.) | 1 star (subjects lost to follow-up unlikely to introduce bias.) | 1 star (complete follow-up – last clinic encounter or death) | 1 star (subjects lost to follow-up unlikely to introduce bias.) | 1 star (subjects lost to follow-up unlikely to introduce bias.) |
